# INPP4B restrains cell proliferation and metastasis via regulation of the PI3K/AKT/SGK pathway

**DOI:** 10.1111/jcmm.13595

**Published:** 2018-03-07

**Authors:** Ying Chen, Zeyu Sun, Mei Qi, Xiao Wang, Weifang Zhang, Chunyan Chen, Juan Liu, Weiming Zhao

**Affiliations:** ^1^ Department of Pathogenic Biology Key Laboratory of Infection and Immunity of Shandong Province School of Basic Medical Sciences Shandong University Jinan Shandong China; ^2^ Department of Pathology School of Basic Medical Sciences Shandong University Jinan Shandong China; ^3^ Department of Hematology Qilu Hospital Shandong University Jinan China

**Keywords:** AKT, cervical cancer, INPP4B, SGK3

## Abstract

Cervical cancer continues to be among the most frequent gynaecologic cancers worldwide. The phosphoinositide 3‐kinase (PI3K)/protein kinase B (AKT) pathway is constitutively activated in cervical cancer. Inositol polyphosphate 4‐phosphatase type II (INPP4B) is a phosphoinositide phosphatase and considered a negative regulatory factor of the PI3K/AKT pathway. INPP4B has diverse roles in various tumours, but its role in cervical cancer is largely unknown. In this study, we investigated the role of INPP4B in cervical cancer. Overexpression of INPP4B in HeLa, SiHa and C33a cells inhibited cell proliferation, metastasis and invasiveness in CCK‐8, colony formation, anchorage‐independent growth in soft agar and Transwell assay. INPP4B reduced the expression of some essential proteins in the PI3K/AKT/SGK3 pathway including p‐AKT, p‐SGK3, p‐mTOR, phospho‐p70S6K and PDK1. In addition, overexpression of INPP4B decreased xenograft tumour growth in nude mice. Loss of INPP4B protein expression was found in more than 60% of human cervical carcinoma samples. In conclusion, INPP4B impedes the proliferation and invasiveness of cervical cancer cells by inhibiting the activation of two downstream molecules of the PI3K pathway, AKT and SGK3. INPP4B acts as a tumour suppressor in cervical cancer cells.

## 1 INTRODUCTION

Despite widespread screening programs, cervical cancer remains frequent cancer among women worldwide, with the highest mortality rates in developing countries.^1^ Although human papillomavirus (HPV) vaccines are available, cervical cancer still causes a considerable amount of death because of the limited application of the vaccine. Persistent infection with high‐risk HPV is the main aetiological factor in cervical carcinogenesis.^2^, ^3^, ^4^ However, HPV infection alone does not sufficiently explain the occurrence of cervical cancer because HPV oncogenes E6 and E7 can immortalize but do not transform human epithelial cells.^5^, ^6^ Thus, HPV infection can be considered an initial hit in the multistep carcinogenesis that leads to cervical cancer, and additional factors are involved in the progression of HPV‐infected lesions to cancer.^7^ The changes in level of some cellular proteins that are crucial regulators in some molecular signalling pathways, as the second hit, might be implicated in this tumorigenic model.

Inositol polyphosphate 4‐phosphatase type II (INPP4B) is a phosphoinositide phosphatase that dephosphorylates PI(3,4)P2 to PI(3)P. Interaction of PI(3,4)P2 with the pleckstrin homology (PH) domain of AKT is required for dimerization and full activation of AKT,^8^ so like phosphatase and tensin homolog (PTEN), INPP4B may act as a tumour suppressor by antagonizing the PI3K/AKT signalling pathway.^9^ However, recent reports demonstrated the controversial role of INPP4B in carcinogenesis in various tumours. INPP4B level was lower in follicular‐like thyroid cancer tissues, melanocytic neoplasm, prostate cancer and breast cancer than in corresponding normal tissues, and knock‐down of INPP4B activated AKT and increased the migratory, invasive and proliferative capacity in these cancer cells.^9^, ^10^, ^11^, ^12^ In contrast, INPP4B was found as an oncogenic regulator in colon cancer, acute myeloid leukaemia and in a subset of melanomas.^13^, ^14^, ^15^ INPP4B promoted cell proliferation and chemical resistance in these cancer cells, mainly by activating serum‐ and glucocorticoid‐regulated kinase 3 (SGK3).^13^, ^15^ Therefore, the role of INPP4B needs to be explored in other cancer models for an overall picture of its function.

Our previous study demonstrated liver kinase B1 (LKB1) as an important tumour suppressor in cervical cancer. Overexpression of LKB1 inhibited HeLa cell proliferation and activated the AMPK pathway.^16^ More importantly, overexpression of LKB1 up‐regulated INPP4B and reduced p‐AKT level, which suggested that LKB1 is involved in negative regulation of the PI3K/AKT pathway by INPP4B.

In this study, we further assessed the function of INPP4B in cervical cancer. INPP4B was overexpressed in cervical cancer cells to explore the role of INPP4B in cell proliferation and metastasis. Our findings confirmed INPP4B as a tumour suppressor in cervical cancer by regulating AKT and SGK3 activities in various cervical cancer cell lines.

## 2 MATERIALS AND METHODS

### 2.1 Cell lines

Cervical cancer cell lines HeLa, SiHa and C33a from the American Type Culture Collection (Manassas, Va) were maintained in DMEM (Invitrogen, Carlsbad, CA) supplemented with 10% foetal calf serum (Invitrogen), 100 IU/mL penicillin and 100 μg/mL streptomycin (Sigma, St. Louis, MO). CaSki cells were maintained in RPMI1640 (Invitrogen, Carlsbad, CA) supplemented with 10% foetal calf serum (Invitrogen). Cells were passaged every 2 days with 0.25% trypsin (Invitrogen).

### 2.2 Plasmids and viral infection

INPP4B complementary DNA was cloned into a lentiviral vector GV367, and lentivirus carrying GV367‐INPP4B or empty vector was packaged by Genechem Co. (Shanghai). Purified lentivirus was used to infect cultured cervical cancer cells in the presence of 5 μg/mL Polybrene (Genechem, China) according to the technical manual. At 72 hour after infection, stable clones were selected with 2 μg/mL puromycin (Thermo Fisher).

### 2.3 Small interfering RNA and transfection

CaSki cells were seeded in 6‐well plates at 4 × 10^5^ cells per well and cultured in medium without antibiotics for 24 hour. INPP4B‐specific siRNA (RiboBio, China) or negative control (NC) was transfected into cells with use of Lipofectamine 2000 (Invitrogen) according to the manufacturer's instructions.

### 2.4 RNA extraction, cDNA synthesis and real‐time PCR

Total RNA in tissue specimens was extracted using TRIzol reagent (Invitrogen) as the manufacturer recommended. Then, cDNA was synthesized with use of the PrimeScript RT reagent kit with gDNA Eraser (Takara, Shiga, Japan) as the manufacturer recommended. The primers were for INPP4B, 5′‐GCCGACCACATCACCACAG‐3′(forward) and 5′‐ TTTCCGCTCACACTTTCCG‐3′(reverse); β‐actin, 5′‐CCATATGCTCACTCAGATGATGT‐3′(forward) and 5′‐GTGTATCATCTCCACAGAGAGTT‐3′ (reverse). Real‐time PCR involved use of SYBR Premix Ex Taq (Takara) following the manufacturer's instructions in the CFX96 Touch Real‐Time PCR Detection System (Bio‐Rad, USA).

### 2.5 Western blot assay

Total protein was extracted from cell lines using RIPA Lysis Buffer (Beyotime Biotechnology, China) as the manufacturer recommended and measured using Pierce BCA Protein Assay Kit (Thermo Fisher, Massachusetts). Western blot assay was performed as described^17^ with specific antibodies for INPP4B (1:1000), PTEN (1:1000), AKT (1:1000) and phospho‐AKT (Ser473, 1:2000, all Cell Signaling Technology); SGK3 (1:1000, Santa Cruz Biotechnology); phospho‐SGK3 (Thr320, 1:1000), mammalian target of rapamycin (mTOR; 1:1000), phospho‐mTOR (1:1000) and phospho‐p70S6k (1:1000, all Cell Signaling Technology); and Phosphoinositide‐dependent kinase‐1 (PDK1; 1:1000) and GAPDH (1:3000, Proteintech, China). GAPDH was a loading control.

### 2.6 Cell proliferation, colony formation and anchorage‐independent growth assays

For cell proliferation assay, cells were seeded onto 96‐well plates at 3 × 10^3^ cells per well. Cell proliferation was determined by use of the Cell Counting Kit‐8 (CCK‐8) (Dojindo, Japan) as the manufacturer recommended. For colony formation assay, cells were cultured on 6‐well plates at 500 cells per well for 10‐14 days, and then colonies were counted after fixing and staining with methyl alcohol and 0.5% crystal violet solution, respectively. Anchorage‐independent growth assays were performed as described.^17^

### 2.7 Cell migration and invasion assays

Transwell inserts (8 μm, 24‐well format; Corning) and Matrigel (BD Bioscience)‐coated Transwell inserts were used to measure cell migration and invasion. In total, 2 × 10^4^ cervical cancer cells in 0.2 mL serum‐free medium were added in the upper chamber of 24‐well plates, with 0.6 mL DMEM containing 20% foetal bovine serum added to the lower chamber. After incubation for 24 to 48 hour, non‐invading cells were removed with use of a cotton swab, and the remaining cells were fixed, stained with 0.5% crystal violet solution and photographed in 5 random fields under a microscope.

### 2.8 Xenograft tumour growth assay

All procedures involving mice were approved by the Institutional Animal Care and Use Committee of Shandong University. A total of 1 × 10^7^ cells were suspended in 200 μL phosphate‐buffered saline and injected subcutaneously into the armpit of 5‐ to 6‐week‐old female BALB/c nu/nu mice (Vital River Laboratories, Beijing). After 2 weeks, tumours were measured using a vernier caliper twice a week for 6 weeks. Tumour volumes were calculated as *V* (mm^3^) = 0.5 × ab^2^. At the end of the assay, green fluorescent protein (GFP; carried by the lentiviral vector GV367) was measured in images from live mice by use of the In‐vivo Imaging System Kodak 2000 imager (Kodak). Then, mice were killed by cervical dislocation under sodium pentobarbital (50 mg/kg) anaesthesia, and tumours were excised and weighed.

### 2.9 Patients and tissue samples

Ethics approval was obtained from the Institutional Research Ethics Committee of Shandong University for all human tissues, cognate clinicopathological data and the experimental protocol used in this study. We used 45 quick‐frozen samples (25 squamous cell cervical cancer tissues and 20 normal cervical tissues) for mRNA extraction and real‐time PCR as well as 217 paraffin‐embedded tissue blocks (100 squamous carcinoma of the cervix, 49 cervical adenocarcinoma and 68 normal cervical tissues) for immunohistochemistry staining. All samples were collected from Qilu Hospital of Shandong University. Normal cervical tissues were from patients with chronic cervicitis or uterine fibroids who had undergone total hysterectomy. Patient consent was obtained for use of the tissue. Diagnosis was based on the World Health Organization Classification of Tumors.

### 2.10 Immunohistochemistry (IHC)

IHC involved the streptavidin‐peroxidase‐biotin IHC method according to standard procedures as we previously described.^17^ Slides were incubated with primary antibody for INPP4B (1:150; Cell Signaling) overnight at 4°C. All stained slides were observed by 2 independent investigators.

### 2.11 Statistical analysis

Data are expressed as mean ± SEM. INPP4B mRNA levels were compared by Student's *t* test. Clinicopathological characteristics of patients with cervical cancer and INPP4B status were assessed by Pearson chi‐square test. Foci formation, anchorage‐independent growth, cell proliferation and cell invasion were analysed by Student's *t* test and one‐way ANOVA. All analysis involved use of SPSS v22.0 (SPSS Inc., Chicago, IL). *P* < .05 was considered statistically significant.

## 3 RESULTS

### 3.1 INPP4B restrains the growth of cervical cancer cells

**Figure 1:**
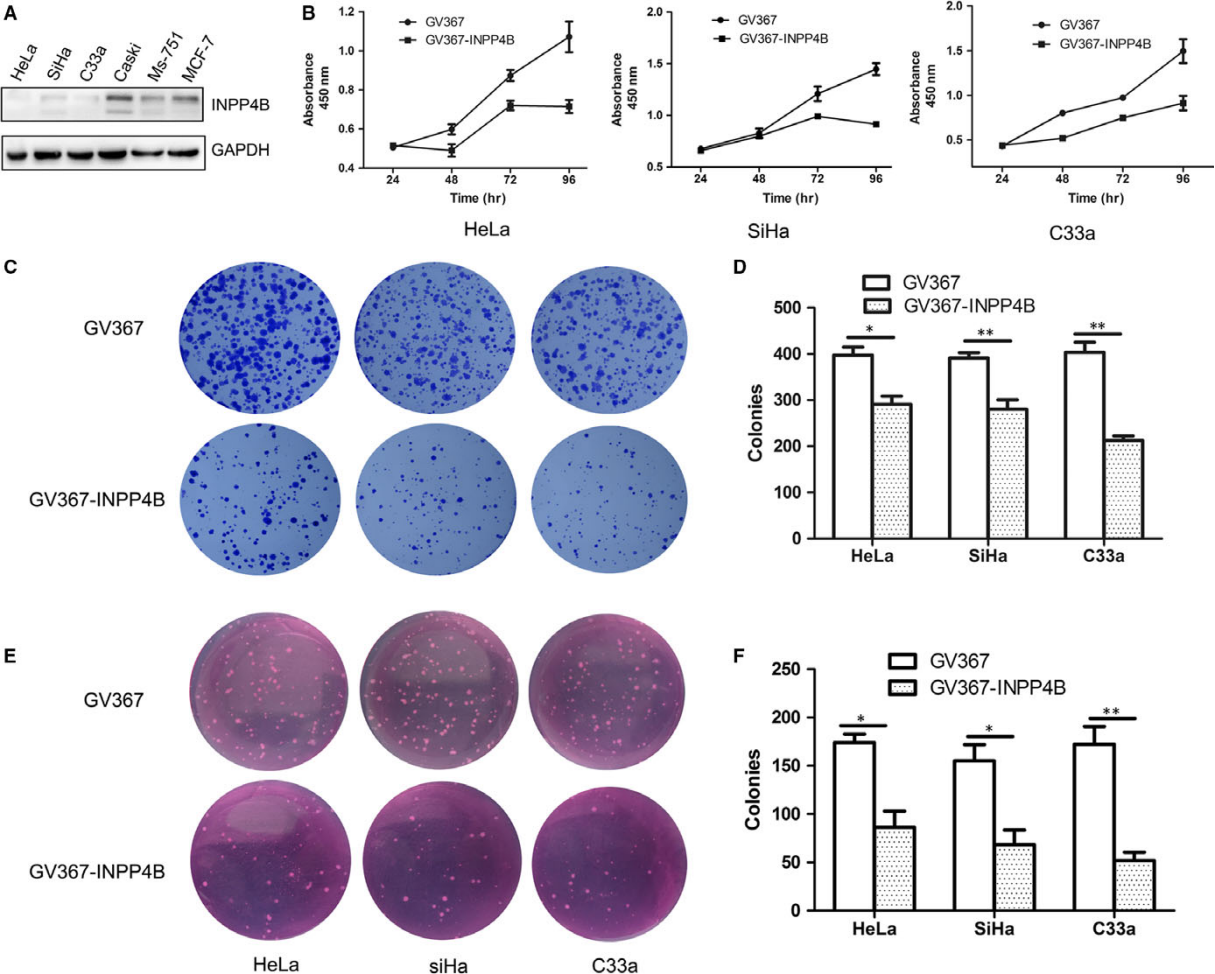
INPP4B overexpression impedes the growth and proliferation of cervical cancer cells. A, Western blot analysis of INPP4B protein level in several cervical cancer cells. B, CCK8 assay analysis of proliferation of cervical cancer cells (HeLa, SiHa and C33a) after transfection with INPP4B cDNA cloned into a lentiviral vector GV367 (GV367‐INPP4B) or empty vector (GV367). *P < .05. C‐D, Colony formation assays of proliferation of cervical cancer cells (HeLa, SiHa and C33a in each column) after transfection of GV367‐INPP4B or empty vector GV367. *P < .05. E‐F, Anchorage‐independent growth assay of proliferation of cervical cancer cells (HeLa, SiHa and C33a in each column) after transfection with GV367‐INPP4B or empty vector GV367. *P < .05, **P < .01. Data are mean ± SEM, n = 3

Firstly, we detected INPP4B in several cervical cancer cell lines with breast cancer MCF‐7 cells used as a positive control. INPP4B was potently expressed in CaSki and Ms‐751 cells as shown by bands near 110 kDa and additional bands of ~85 kDa, which was previously reported in melanocytic cells,^10^ but was almost absent in HeLa, SiHa and C33a cells (Figure 1A). Then, we used HeLa, SiHa and C33a cells with stable expression of INPP4B by transfection of a lentivirus carrying the protein gene. On CCK‐8 assay, proliferation was less for the three cell lines with exogenous expression of INPP4B than control cells (Figure 1B, All *P* < .01). As compared with controls, cells with stable INPP4B overexpression for 14 days showed significantly decreased colony formation: HeLa, 397.3 ± 17.48 vs 291 ± 17.62, *P* = .0128; SiHa, 391.0 ± 11.59 vs 280.3 ± 20.42, *P* = .0092; and C33a, 403.3 ± 21.67 vs 213.3 ± 9.21, *P* = .0013 (Figure 1C and D). Moreover, INPP4B expression reduced cell growth of cells in soft agar: HeLa, 170.7 ± 11.85 vs 86.33 ± 16.59, *P* = .0144; SiHa, 155.0 ± 17.04 vs 68.33 ± 15.06, *P* = .0189; and C33a, 172.3 ± 18.41 vs 52.0 ± 8.54, *P* = .0041 (Figure 1E and F).

### 3.2 INPP4B impedes migration and invasion of cervical cancer cells

**Figure 2:**
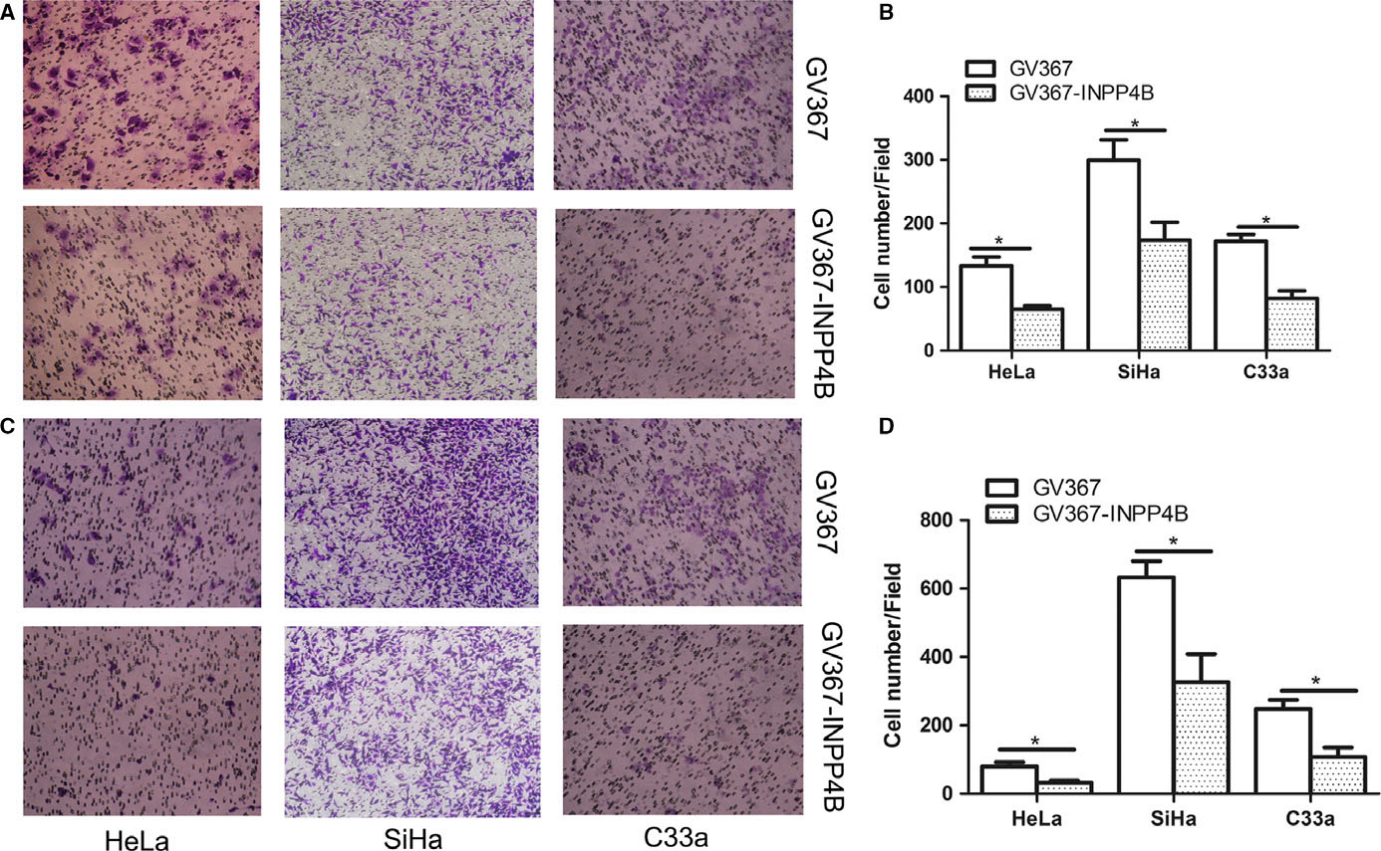
INPP4B overexpression reduces the migration and invasion of cervical cancer cells. A‐B, Transwell assay of migration ability of cervical cancer cells (HeLa, SiHa and C33a in each column) after transfection with GV367‐INPP4B and empty vector GV367. *P < .05. C‐D, Transwell invasion assay of invasion ability of INPP4B stably transfected cervical cancer cells (HeLa, SiHa and C33a in each column) and control cells. *P < .05. Data are mean ± SEM, n = 3

We further explored the role of INPP4B in the migration and invasion ability of cervical cancer cells by Transwell assay. Overexpression of INPP4B notably reduced the number of migrated cells as compared with controls: HeLa, 133.0 ± 14.22 vs 65.0 ± 5.57, *P* = .0112; SiHa, 299.7 ± 31.78 vs 173.7 ± 28.09, *P* = .0411; and C33a, 172.0 ± 10.79 vs 82.0 ± 12.22, *P* = .0053 (Figure 2A and B). Moreover, the number of invasive cells was decreased: HeLa, 79.67 ± 12.44 vs 32.0 ± 6.658; SiHa, 633.0 ± 47.61 vs 326.0 ± 82.44; C33a, 247.7 ± 26.60 vs 107.7 ± 27.44 (all *P* < .05) (Figure 2C and D).

### 3.3 INPP4B suppresses the phosphorylation of AKT and SGK3

**Figure 3:**
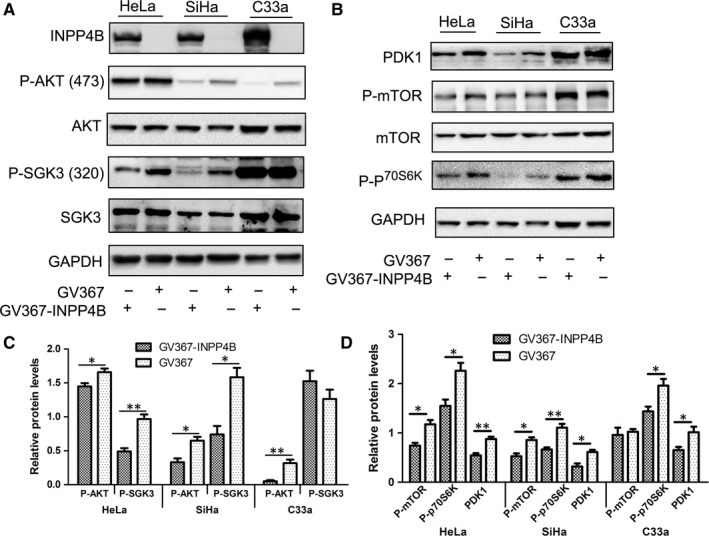
INPP4B suppresses the phosphorylation of AKT and SGK3 in cervical cancer cells. A, Western blot assay of phosphorylation of AKT and SGK3 in cervical cancer cells (HeLa, SiHa and C33a) after transfection with GV367‐INPP4B or empty vector GV367. GAPDH was the loading control. B, Western blot assay of protein expression of PDK1 and several downstream molecules of AKT/SGK3. GAPDH was the loading control. C‐D Quantification of protein expression. Data are mean ± SEM, n = 3; *P < .05, **P < .01

To understand the mechanisms by which INPP4B reduced the proliferation and invasion of cervical cancer cells, we analysed the expression and activation of several pivotal molecules in the PI3K/AKT pathway. Consistent with other studies, phospho‐AKT (Thr473) level was lower in HeLa, SiHa and C33a cells with INPP4B expression than controls (Figure 3A and C). Phospho‐SGK3 (Thr320) level was reduced in INPP4B‐expressing HeLa and SiHa cells (Figure 3A and C). This result does not agree with previous reports, which depicted INPP4B as a tumour promoter by activating SGK3.^13^, ^15^ Considering that the activation of SGK3 and AKT mainly depends on their upstream molecule PDK1, we measured the levels of PDK1 and several downstream molecules of AKT/SGK3. Molecules downstream of AKT/SGK3, including mTOR/phospho‐mTOR and phospho‐p70S6K, were reduced in INPP4B‐expressing HeLa and SiHa cells (Figure 3B and D) as was the expression of the upstream molecule PDK1.

### 3.4 Depletion of INPP4B promotes proliferation of CaSki cells

**Figure 4:**
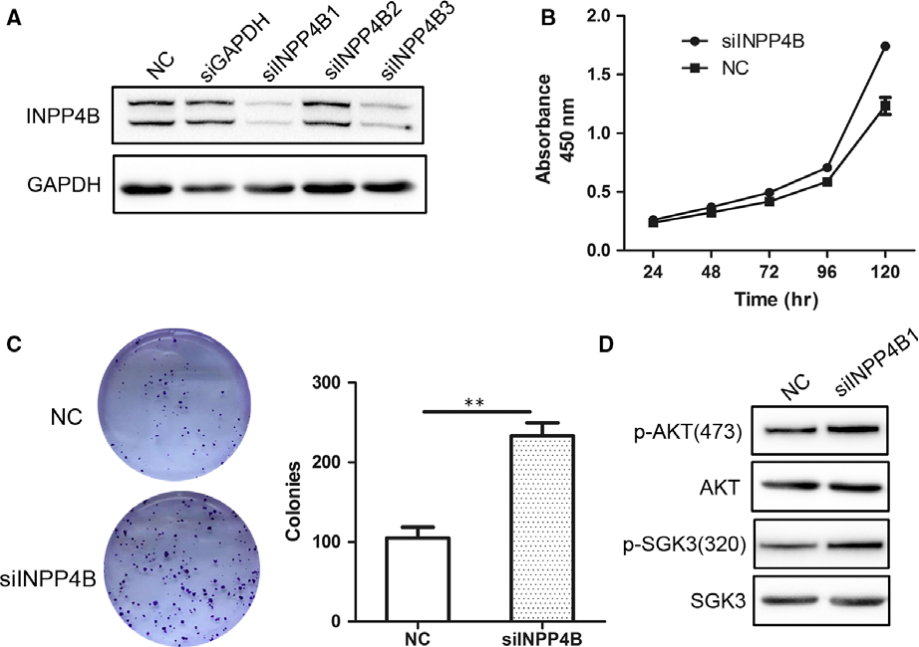
INPP4B knock‐down promotes proliferation of CaSki cells by elevation of p‐AKT and p‐SGK3. A, Western blot assay of INPP4B in CaSki cells with INPP4B‐specific siRNA (siINPP4Bs) or control siRNA (NC). B, CCK‐8 assay of CaSki cells with siINPP4B1 or NC. **P < .01. C, Colony formation assay of CaSki cells with siINPP4B1 or NC. **P < .01. Data are mean±SEM, n = 3. D, Western blot assay of AKT and SGK3 in CaSki cells with INPP4B‐specific siRNA (siINPP4Bs) or control siRNA (NC)

Effect of three INPP4B‐specific siRNA for INPP4B depletion in CaSki cells was measured by Western blot analysis (Figure 4A). We used siINPP4B 1 in following experiments because of its efficient knock‐down of INPP4B expression. In agreement with our findings in other cervical cancer cells, cell proliferation was obviously higher with siINPP4B than control treatment, as detected by CCK‐8 and colony formation assay (Figure 4B and C). More importantly, INPP4B depletion increased the phosphorylation of both AKT (Thr 473) and SGK3 (Thr320) (Figure 4D).

### 3.5 INPP4B overexpression inhibits tumour growth in vivo

**Figure 5:**
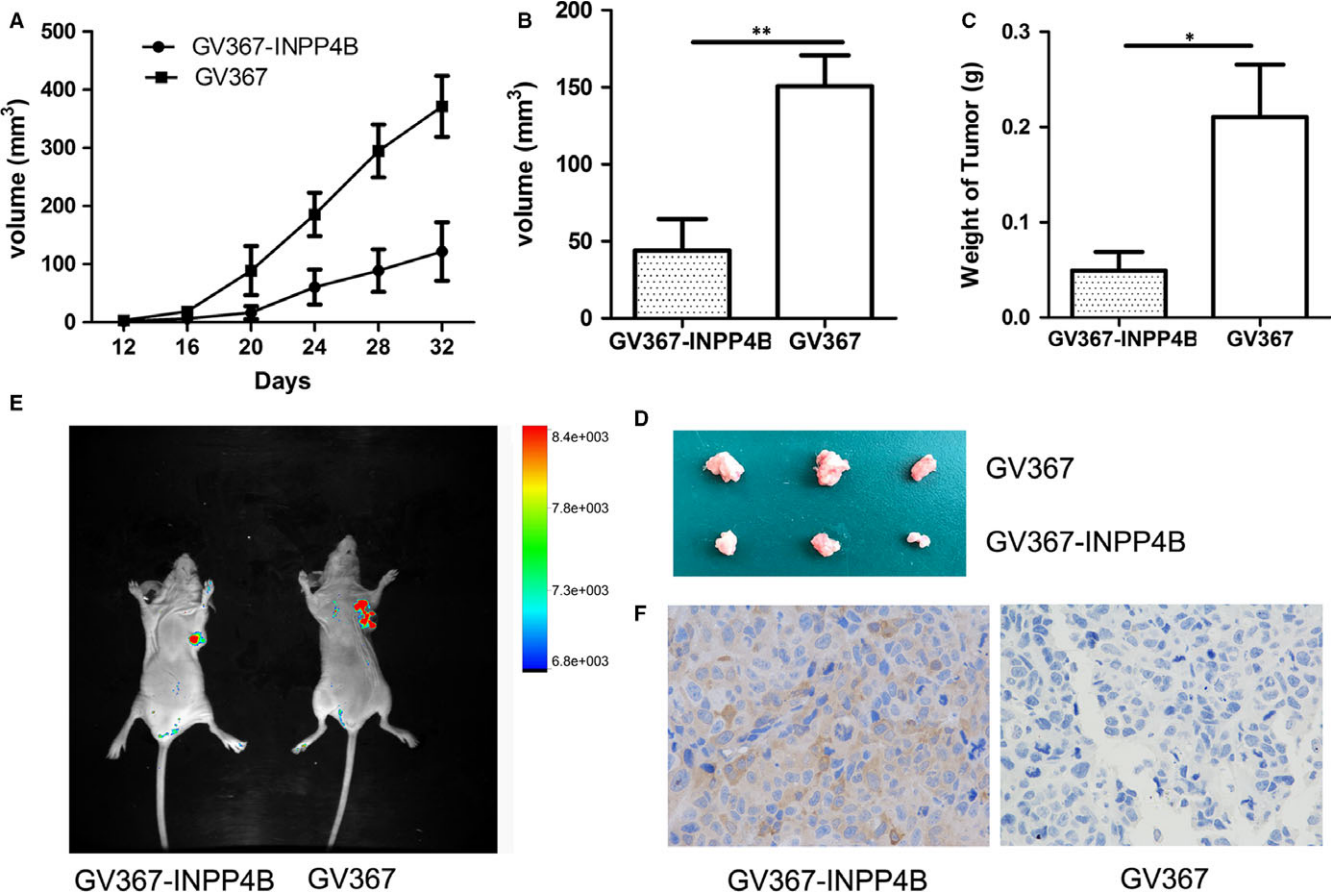
Overexpression of INPP4B inhibits the growth of cervical cancer cells in mice in vivo. A, Growth curve of xenografts of HeLa cells transfected with GV367‐INPP4B or negative control GV367 in nude mice. Tumour diameters were measured every 4 days. n = 20, **P < .01. B‐C, Volume and weight of harvested xenografts of HeLa cells transfected with GV367‐INPP4B or negative control GV367. Data are mean ± SEM, n = 20. *P < .05, **P < .01. D, Tumours excised from mice. Top: mice with HeLa control cells, bottom: mice with HeLa‐INPP4B cells. E Fluorescent signals of green fluorescent protein (GFP) in mouse tumours after injection of cells with GV367‐INPP4B or vector GV367. F, Immunohistochemistry staining of fluorescent signals of INPP4B in tumours after injection of cells with GV367‐INPP4B or vector GV367

To evaluate whether INPP4B suppresses the growth of cervical cancer cells in vivo, we subcutaneously injected HeLa cells stably expressing INPP4B into the armpit of nude mice. Nodules were found in the armpit of mice at 2 weeks after inoculation. At 32 days post‐injection, the tumour size with HeLa‐INPP4B and control injection was 371.3 ± 52.4 vs 121.8 ± 50.46 mm^3^ (Figure 5A, *P* = .0083). The volume and weight of tumours excised were lower with HeLa‐INPP4B than control injection [volume: 44.00 ± 20.40 vs 150.7 ± 20.02, *P* = .0039; weight: 0.04933 ± 0.01963 vs 0.2107 ± 0.05495, *P* = .02; Figure 5B‐D]. Consistently, GFP fluorescent signals were more extensive in tumours with control than HeLa‐INPP4B injection (Figure 5E). IHC staining verified the presence of INPP4B in the tumours with HeLa‐INPP4B injection (Figure 5F).

### 3.6 Expression of INPP4B protein in cervical tissues

**Figure 6:**
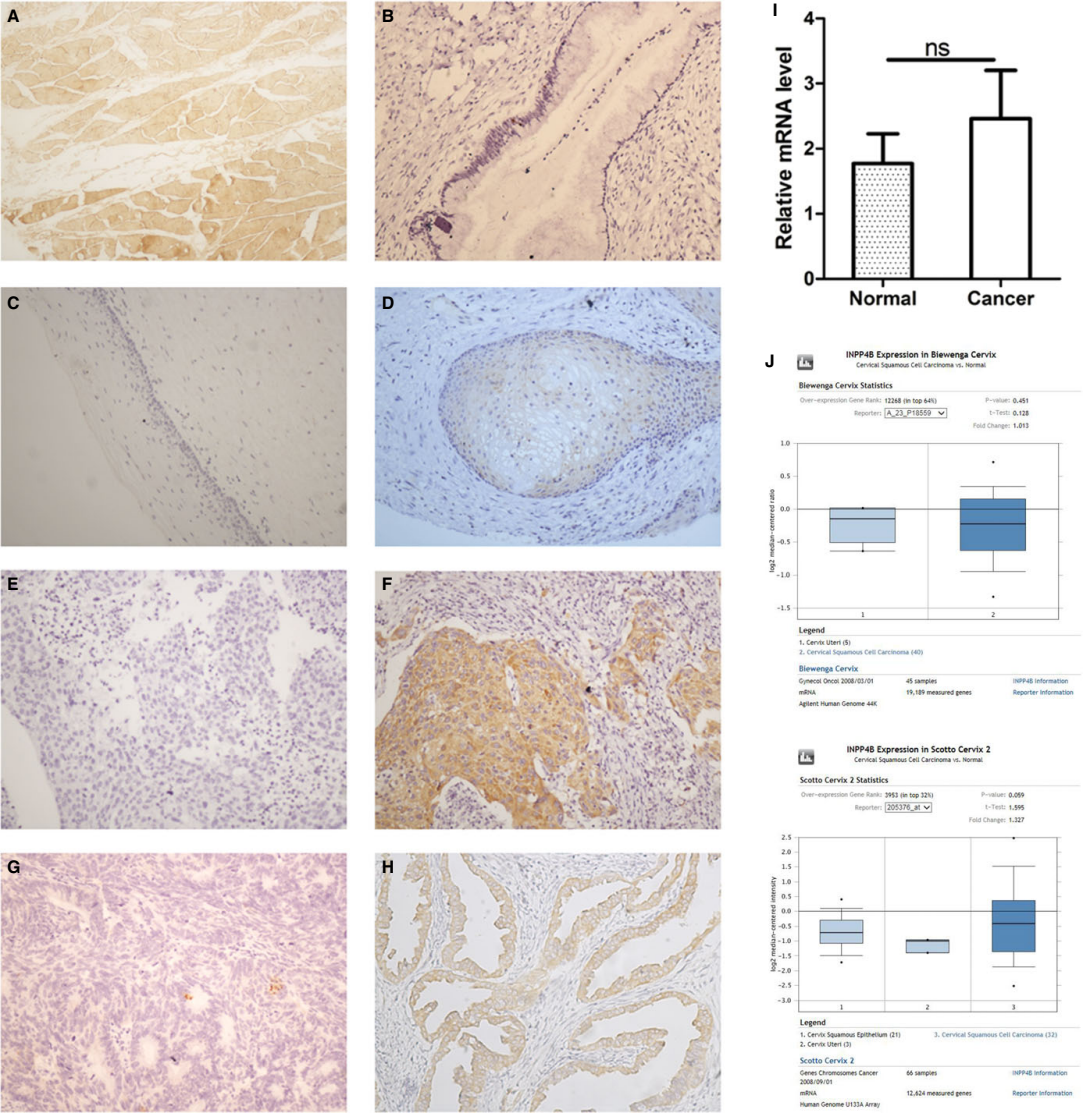
INPP4B protein expression in cervical tissues and cervical cancer cell lines. A, Normal human skeletal muscles, B, normal cervical columnar epithelium (negative), C, normal cervical squamous epithelium (negative), D, normal cervical squamous epithelium (cytoplasm), E, cervical squamous cell carcinoma (negative), F, cervical squamous cell carcinoma (cytoplasm), G, cervical adenocarcinoma (negative), H, cervical adenocarcinoma (cytoplasm) and I, mRNA levels of INPP4B in normal and cervical cancer tissues. Data are mean ± SEM, n = 45. ns, not significant. J, mRNA levels of INPP4B in cervical tissues in Oncomine database

We detected INPP4B in normal and cervical cancer tissues by IHC, with normal skeletal muscle tissues as a positive control (Figure 6A). Among the normal cervical tissues, 20 of 62 squamous cell epithelial samples showed cytoplasmic staining for INPP4B, with a positive rate of 32.3%. No positive staining found in all 6 columnar cell epithelial samples (Figure 6B‐D). Among squamous cell cervical carcinoma samples, 39 of 100 (39%) had positive cytoplasmic INPP4B staining and 61 of 100 (61%) negative staining (Figure 6E and F). For cervical adenocarcinoma samples, 15 of 49 (30.6%) had positive INPP4B staining (also largely in the cytoplasm) and 34 of 49 (69.4%) negative staining (Figure 6G and H). INPP4B protein level did not differ between normal and cancer tissues (*P* = .406) and was not associated with age, clinical stage and other clinicopathological characteristics of patients (*P* > .05, data not shown). In agreement with IHC findings, in frozen cervical tissues, INPP4B mRNA level did not differ between normal and cervical cancer samples (Figure 6I, *P* = .4108).

To verify our results, we referenced the data for INPP4B mRNA expression in the Oncomine database. Two available databases, Biewenga and Scotto Cervix, had similar results as we found (Figure 6J).

## 4 DISCUSSION

PI3K/AKT is a crucial pathway in carcinogenesis and is deregulated in various tumours including cervical cancer.^8^, ^18^, ^19^ Mutation data from many independent studies showed mutations in PIK3CA and PTEN in various proportions in cervical cancer (13%‐23% in PIK3CA, 5%‐8% in PTEN).^20^ A recent review found that HPV oncogenes E6/E7/E5 enhance the PI3K/AKT/mTOR signalling pathway by directly activating PI3K and the downstream AKT and p70S6K, which consequently modulates tumour initiation and progression as well as patient outcome in cervical cancer.^21^ As a negative regulatory molecule of the PI3K/AKT pathway, INPP4B has been reported to have tumour suppression effects in various tumours.^9^, ^10^, ^11^, ^12^ In our previous study, we found a tumour suppressor protein, LKB1, which inhibits proliferation and up‐regulates the level of INPP4B protein in cervical cancer cells.^16^ This finding suggests that INPP4B, such as LKB1, may play a suppressive role in cervical cancer. In the present study, we verified that INPP4B impaired cell proliferation, migration and invasive capacity by affecting PI3K/AKT/SGK3 signalling. In addition, INPP4B suppressed tumour growth of xenografted cervical cancer cells in mice.

Our results demonstrated that INPP4B was almost absent in HeLa, SiHa and C33a cells. Ectopic expression of INPP4B inhibited cell proliferation, migration and invasion of cervical cancer cells (Figure 1A‐Figure2D). Knock‐down of INPP4B in CaSki cells confirmed its suppressive effect on cell proliferation (Figure 4B and C). As well, it obviously impaired the tumour formation of INPP4B‐expressed HeLa cells in a nude mice xenograft model (Figure 5A‐D). In line with those of the previous studies in prostate cancer, basal‐like breast cancer and ovary cancer,^11^, ^12^, ^22^ our results reveal the suppressive role of INPP4B in tumorigenesis of cervical cancer.

The review by Woolley et al^23^ summarized the mechanism underlying the complexity in function of INPP4B in cancer. Most of the previous studies identified INPP4B as a suppressor of oncogenic growth of cancer cells via the traditional PI3K/INPP4B/AKT axis, whereby INPP4B dephosphorylates PI(3,4)P2 to obstruct the activation of AKT. In agreement, we found that ectopic expression of INPP4B decreased phospho‐AKT and its downstream molecules in cervical cancer cells (Figure 3A). Nevertheless, some recent reports have indicated that INPP4B acts as an oncogenic driver by activating SGK3 in several kinds of cancer cells.^13^, ^15^, ^24^ As a member of a serine/threonine protein kinase family, SGK3 is highly homologous to and shares substrate specificity to the protein kinase B (PKB)/AKT family.^25^ Tessier et al^25^ reported that the main product of INPP4B, PI(3)P, can bind to a Phox homology domain of SGK3, thereby targeting the protein to endosomes, where PI3K and PDK1 phosphorylate SGK3 at two regulatory sites (Thr‐320 and Ser‐486). The phosphorylation at the two sites induces the full kinase activity of SGK3. We found that overexpression of INPP4B diminished the activity of SGK3 by decreasing its phosphorylation in cervical cancer cells (Figure 3A). The elevation of p‐SGK3 after INPP4B knock‐down in CaSki cells confirmed this result (Figure 4D). We further found that INPP4B reduced the level of an upstream molecule, PDK1, in the PI3K/AKT pathway (Figure 3B). Considering that PDK1 is one of the important kinases to phosphorylate SGK3, it is reasonable that reduced PDK1 expression resulted in the reduced phosphorylation of SGK3. Our results shed new light on the complex mechanism of the paradoxical function of INPP4B in cancer. In different cancers, INPP4B may affect PI3K/AKT/SGK signal output dissimilarly, which contributes to various biological behaviours in those cancer cells.

In our study, INPP4B expression was lost in the majority of cervical carcinomas, which is consistent with INPP4B as a tumour suppressor (Figure 6). As INPP4B inhibits proliferation of cervical cancer in vitro and in vivo, this protein may serve as a possible candidate for adjuvant therapy of cervical cancer. We also found INPP4B was not detected in a significant number of normal cervical tissues. The low expression of INPP4B in a certain proportion of normal cervix is analogous to that of LKB1, a tumour suppressor upstream of INPP4B, which also displays weak or no expression in normal colorectal, pancreas and cervical tissues.^16^, ^26^, ^27^ We speculate that low expression of INPP4B could be due to lack of tumorigenesis‐associated stresses to INPP4B in normal cervical cells.

Cervical cancer is a pathogenic‐related cancer, with long‐term infection of HPV and interactions between the viral oncoproteins E6/E7 and cellular molecules promoting the transformation of cervical cells. We found that INPP4B inhibited the growth of HPV‐negative cells to a larger extent than that of HPV‐positive cells (Figure 1C‐F). Thus, E6/E7 may be involved in the regulatory role of INPP4B. Overexpression of INPP4B did not changed the level of p53 and Rb protein, two target molecules of E6 and E7, in various cervical cancer cells (data not shown). The mechanism of the crosstalk between INPP4B and HPV E6/E7 needs further investigation.

In summary, we have identified INPP4B as a tumour suppressor in cervical cancer. INPP4B impedes the tumorigenic phenotypes of cervical cancer cells by inhibiting the activation of two downstream molecules of the PI3K pathway, AKT and SGK3. In addition, INPP4B expression significantly prevented tumour growth in mice in vivo. Various inhibitors of molecules in the PI3K/AKT pathway are undergoing pre‐clinical validation for many tumour types.^28^, ^29^ INPP4B may also be a potential candidate for adjuvant therapy of cervical cancer and other tumours that are activated in the PI3K pathway.

## CONFLICT OF INTEREST

The authors declare that they have no conflict of interests.
